# Sevoflurane-induced amnesia is associated with inhibition of hippocampal cell ensemble activity after learning

**DOI:** 10.1242/bio.059666

**Published:** 2022-12-21

**Authors:** Akiyo Kameyama, Hirotaka Asai, Masanori Nomoto, Shuntaro Ohno, Khaled Ghandour, Noriaki Ohkawa, Yoshito Saitoh, Mitsuaki Yamazaki, Kaoru Inokuchi

**Affiliations:** ^1^ Department of Biochemistry, Graduate School of Medicine and Pharmaceutical Sciences, University of Toyama, Toyama 930-0194, Japan; ^2^ Department of Anesthesiology, Graduate School of Medicine and Pharmaceutical Sciences, University of Toyama, Toyama 930-0194, Japan; ^3^ Research Center for Idling Brain Science (RCIBS), Department of Biochemistry, Graduate School of Medicine and Pharmaceutical Sciences, University of Toyama, Toyama 930-0194, Japan; ^4^ Core Research for Evolutional Science and Technology (CREST), Japan Science and Technology Agency (JST), University of Toyama, Toyama 930-0194, Japan; ^5^ Precursory Research for Embryonic Science and Technology (PRESTO), JST, Saitama 332-0012, Japan; ^6^ Division for Memory and Cognitive Function, Research Center for Advanced Medical Science, Comprehensive Research Facilities for Advanced Medical Science, Dokkyo Medical University, Tochigi 321-0293, Japan; ^7^ Department of Biochemistry, Faculty of Pharmacy, Cairo University, Cairo, 11562, Egypt

**Keywords:** Amnesia, General anesthesia, Sevoflurane, Hippocampus, Neuronal ensemble, Calcium imaging

## Abstract

General anesthesia could induce amnesia, however the mechanism remains unclear. We hypothesized that suppression of neuronal ensemble activity in the hippocampus by anesthesia during the post-learning period causes retrograde amnesia. To test this hypothesis, two experiments were conducted with sevoflurane anesthesia (2.5%, 30 min): a hippocampus-dependent memory task, the context pre-exposure facilitation effect (CPFE) procedure to measure memory function and *in vivo* calcium imaging to observe neural activity in hippocampal CA1 during context exploration and sevoflurane/home cage session. Sevoflurane treatment just after context pre-exposure session impaired the CPFE memory, suggesting sevoflurane induced retrograde amnesia. Calcium imaging showed sevoflurane treatment prevented neuronal activity in CA1. Further analysis of neuronal activity with non-negative matrix factorization, which extracts neural ensemble activity based on synchronous activity, showed that sevoflurane treatment reduced the reactivation of neuronal ensembles between during context exploration just before and one day after sevoflurane inhalation. These results suggest that sevoflurane treatment immediately after learning induces amnesia, resulting from suppression of reactivation of neuronal ensembles.

## Teaser

**Summary:** This research shows the effect of sevoflurane anesthesia just after learning on amnesia and that sevoflurane inhibits neuronal ensemble activity in hippocampal CA1.

## INTRODUCTION

Anesthesia is indispensable for modern surgical procedures, but it can induce amnesia. Amnesia impairs memory not only during anesthesia but also retrogradely or anterogradely. Retrograde or anterograde amnesia are defined as a loss of memory about events which is acquired before or after the incidents that caused the amnesia. Previous reports show that retrograde or anterograde amnesia occur after general anesthesia in humans (Aulakh et al., 2018; Gruber and Reed, 1968; Koht and Moss, 1997; Quraishi et al., 2007; Sohn et al., 2021; Twersky et al., 1993) and rodents (Dutton et al., 2002, 2001; Gerlai and McNamara, 2000; O'Gorman et al., 1998). However, the underlying mechanism of anesthesia-induced amnesia remains unclear.

The hippocampus is one of the key brain regions involved in memory formation as well as anesthesia-induced amnesia (Perouansky and Pearce, 2011; Scoville and Milner, 1957; Wang and Orser, 2011). General anesthesia impairs neuronal activity in the rodent hippocampus (Perouansky et al., 2010; Yang et al., 2021; Yin et al., 2016). Anesthetics are known to inhibit one of the fundamental mechanisms of learning and memory formation, namely long-term potentiation, in hippocampal CA1 neurons *in vitro* and *in vivo* (Haseneder et al., 2009; Ishizeki et al., 2008; Liu et al., 2018; Nabavi et al., 2014; Squire, 1999). Therefore, general anesthesia can cause amnesia (Aulakh et al., 2018; Perouansky et al., 2010; Yamaji et al., 1989). However, how the hippocampal activity altered by anesthesia causes amnesia remains unclear.

Reactivation of hippocampal neuronal ensembles that are activated during learning is essential for memory consolidation (Ghandour et al., 2019; Kumar et al., 2020; Wilson and McNaughton, 1994). Artificial inactivation of neuronal activity after learning inhibits consolidation and impairs memory performance (Boyce et al., 2016; Clawson et al., 2021; Miyamoto et al., 2016). Memories are stored in subsets of neuronal populations called memory trace which are characterized by the expression of immediate early genes such as cFos and Arc (Denny et al., 2014; Liu et al., 2012; Ohkawa et al., 2015; Tanaka et al., 2014; Yiu et al., 2014). Memory traces in hippocampal CA1 are first activated during learning and then reactivated during post-learning sleep (Ghandour et al., 2019). From these reports, we hypothesized that anesthesia-induced amnesia results from the inhibition of reactivation of hippocampal neuronal ensembles immediately after learning.

To test this hypothesis, we assessed the effect of sevoflurane, a volatile general anesthetic, on memory function using a hippocampus-dependent memory task, context pre-exposure facilitation effect (CPFE) paradigm (Fanselow, 1990; Ghandour et al., 2019; Ohkawa et al., 2015; Rudy and O'Reilly, 2001; Schiffino et al., 2011; Suzuki et al., 2022). In the CPFE paradigm mice have to learn the association of context information presented first with foot shock experience delivered later. In this research, mice were exposed to sevoflurane just after context learning for assessing the effect of sevoflurane on retrograde amnesia. Furthermore, we investigated how anesthesia given immediately after learning affected hippocampal neuronal activity at the ensemble level using *in vivo* calcium (Ca^2+^) imaging (Asai et al., 2020; Barretto et al., 2011; Ghandour et al., 2019; Ghosh et al., 2011; Kitamura et al., 2015; Ziv et al., 2013).

## RESULTS

### Sevoflurane treatment immediately after context pre-exposure induced amnesia in CPFE paradigm

**Fig. 1.**
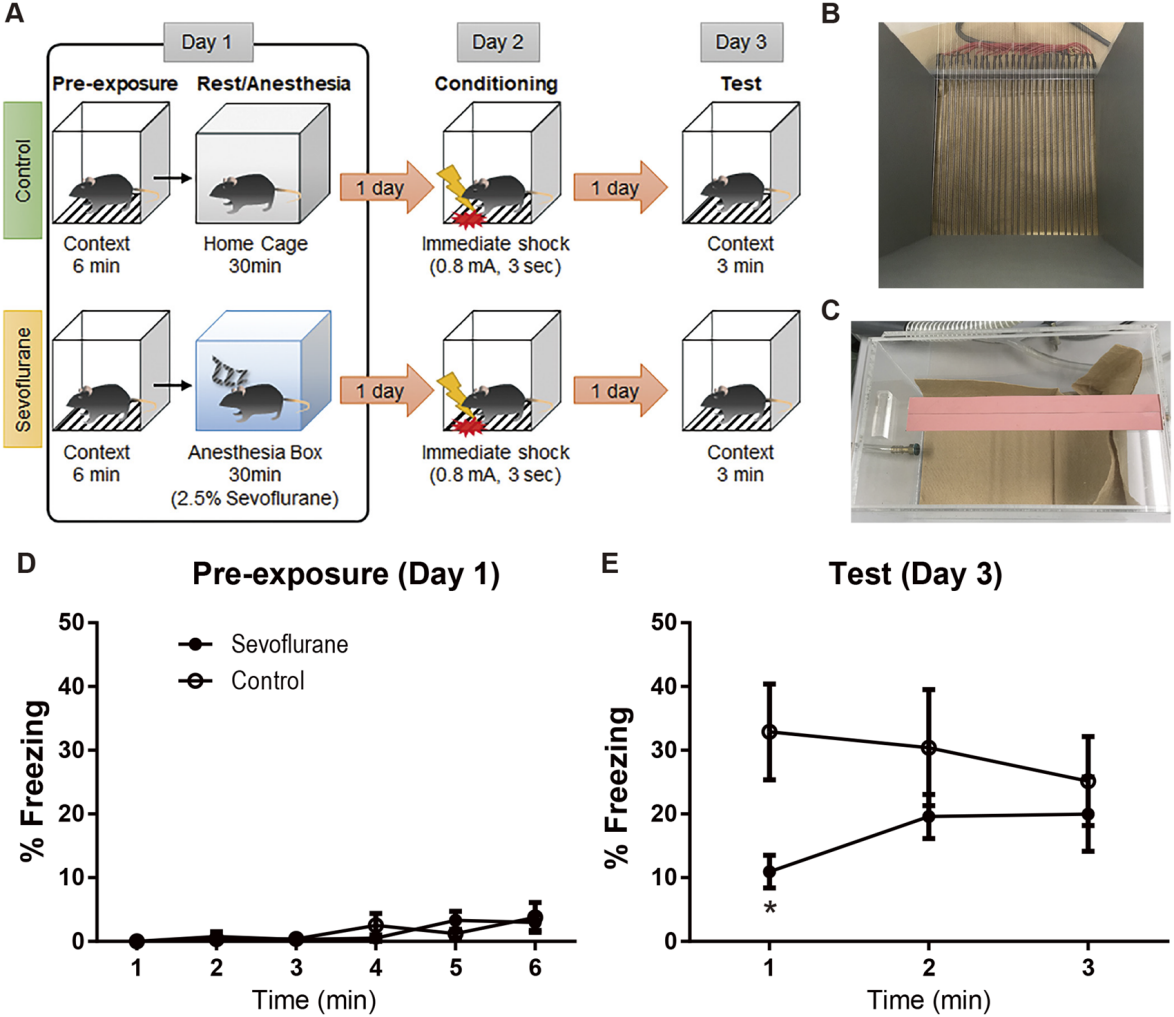
**Sevoflurane treatment induced amnesia in the context pre-exposure facilitation effect (CPFE) paradigm.** (A) Schematic of the procedure and sevoflurane treatment. (B,C) Context and anesthesia boxes. (D,E) The levels of freezing response on Day 1 pre-exposure (D) and Day 3 test session (E). Statistical values from Bonferroni's multiple-comparison test are provided in Table S1. *n* = 8 non-anesthesia control mice, 11 sevoflurane treatment mice. *P < 0.05. Data are shown as means ± s.e.m.

To investigate the effect of anesthesia on retrograde amnesia, we used sevoflurane treatment combined with a hippocampus-dependent memory task: the CPFE paradigm (Ghandour et al., 2019; Ohkawa et al., 2015; Rudy and O'Reilly, 2001; Schiffino et al., 2011; Suzuki et al., 2022). In this paradigm (Fig. 1A), mice associated context information (Day 1 pre-exposure; Fig. 1B) with foot shock information (Day 2 conditioning) when they received a foot shock immediately after entering a previously encountered context in the Day 1 pre-exposure session. The anesthesia group of mice was treated with sevoflurane for 30 minutes in an anesthesia box (Fig. 1C) immediately after pre-exposure to the context, while the control group was returned to their home cage. During pre-exposure session on Day 1, no difference was observed in behavioral activity between both groups (Fig. 1D). During the test session on Day 3, the mice in the sevoflurane group showed significantly less freezing than mice in the control group. (Fig. 1E; *n*=8 control mice, 11 sevoflurane anesthesia mice. Statistical values from Bonferroni's multiple-comparisons test are provided in Table S1). These results shows that the post-learning sevoflurane treatment induces amnesia in hippocampal dependent memory task, which is likely attributable to the inhibitory effect of sevoflurane on memory consolidation.

### Sevoflurane suppressed neuronal activity in hippocampal CA1

**Fig. 2.**
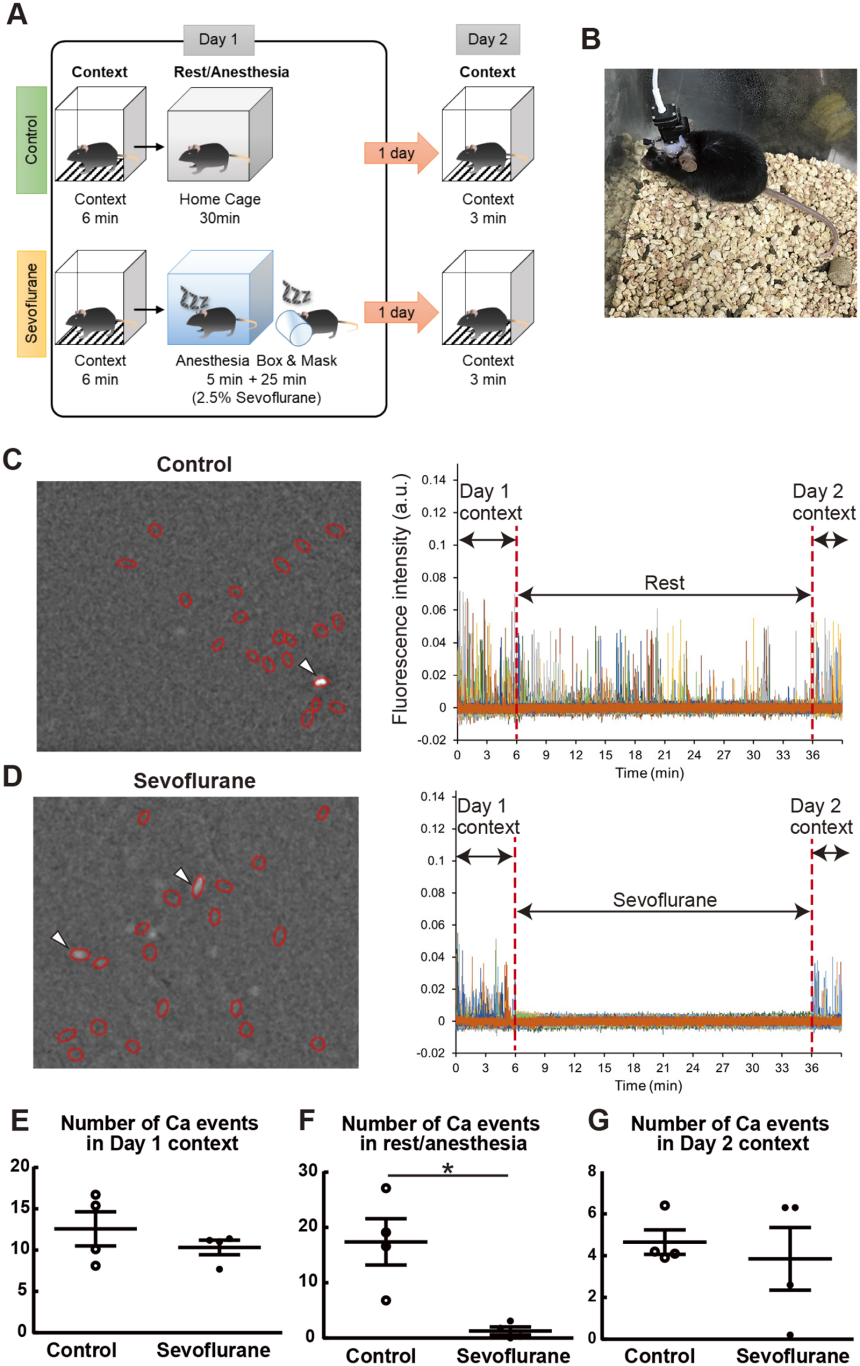
**Sevoflurane treatment suppressed neuronal activity in hippocampal CA1.** (A) Schematic of Ca^2+^ imaging experiment and sevoflurane treatment. (B) Imaging of neuronal activity in home cage. (C,D) Representative images of 20 detected cells (red circles, left) and Ca^2+^ transients (right). Arrow heads mean active neurons in this example frame. (E-G) The number of Ca^2+^ events in each session: (E) Day 1 context session, (F) Rest/sevoflurane treatment session, (G) Day 2 context session. (E) Non-anesthesia control mice (12.58 ± 2.065) versus sevoflurane treatment mice (10.33 ± 0.879), 95% confidence interval (CI) = −7.740 to 3.240; *F*_(3,3)_ = 5.519, *P* = 0.1943; t_6_ = 1.003, *P* = 0.3547, unpaired *t*-test. (F) Non-anesthesia control mice (17.40 ± 4.183) versus sevoflurane treatment mice (1.300 ± 0.7176), 95% CI = −29.19 to −3.008; *F*_(3,3)_ = 33.98, *P* = 0.0163; t_3.176_ = 3.793, *P* = 0.0291, unpaired *t*-test with Welch's correction. (G) Non-anesthesia control mice (4.650 ± 0.587) versus sevoflurane treatment mice (3.580 ± 1.497), 95% CI = −4.734 to 3.134; *F*_(3,3)_ = 6.511, *P* = 0.1582, t_6_ = 0.4976, *P* = 0.6365, unpaired *t*-test. *P < 0.05. Data are shown as means ± s.e.m.

To investigate the effect of sevoflurane on neuronal activity in hippocampal CA1, we conducted Ca^2+^ imaging in freely moving mice during context exploration and sevoflurane anesthesia (Fig. 2A). Calcium imaging was performed for three sessions: context exposure (Day 1 context, 6 min), rest in home cages or sevoflurane anesthesia (rest/sevoflurane, 30 min) and context re-exposure (Day 2 context, 3 min). To visualize neuronal activity, the Thy1-G-CaMP7 mice were fitted with a head-mount miniaturized microscope nVista^™^ (Aly et al., 2022; Asai et al., 2020; Barretto et al., 2011; Ghandour et al., 2019; Ghosh et al., 2011; Kitamura et al., 2015) as shown in Fig. 2B. These mice express G-CaMP7, a genetically encoded Ca^2+^ indicator, in pyramidal neurons located in the deep layer of hippocampal CA1 (Sato et al., 2018 preprint). Representative images of 20 detected cells (left) and Ca^2+^ transients (right) in both groups were shown in Fig. 2C and D. Both groups of mice showed similar Ca^2+^ activity during the Day 1 context (Fig. 2E), but the mice during sevoflurane inhalation had significantly lower neuronal activity than the mice during rest session in control group (Fig. 2F). There was no significant difference between two groups during Day 2 context (Fig. 2G). These data suggest that sevoflurane suppressed the number of Ca^2+^ events in hippocampal CA1 during administration, but did not influence on it at the next day.

### Sevoflurane suppressed reactivation of neuronal ensembles during context exploration

**Fig. 3.**
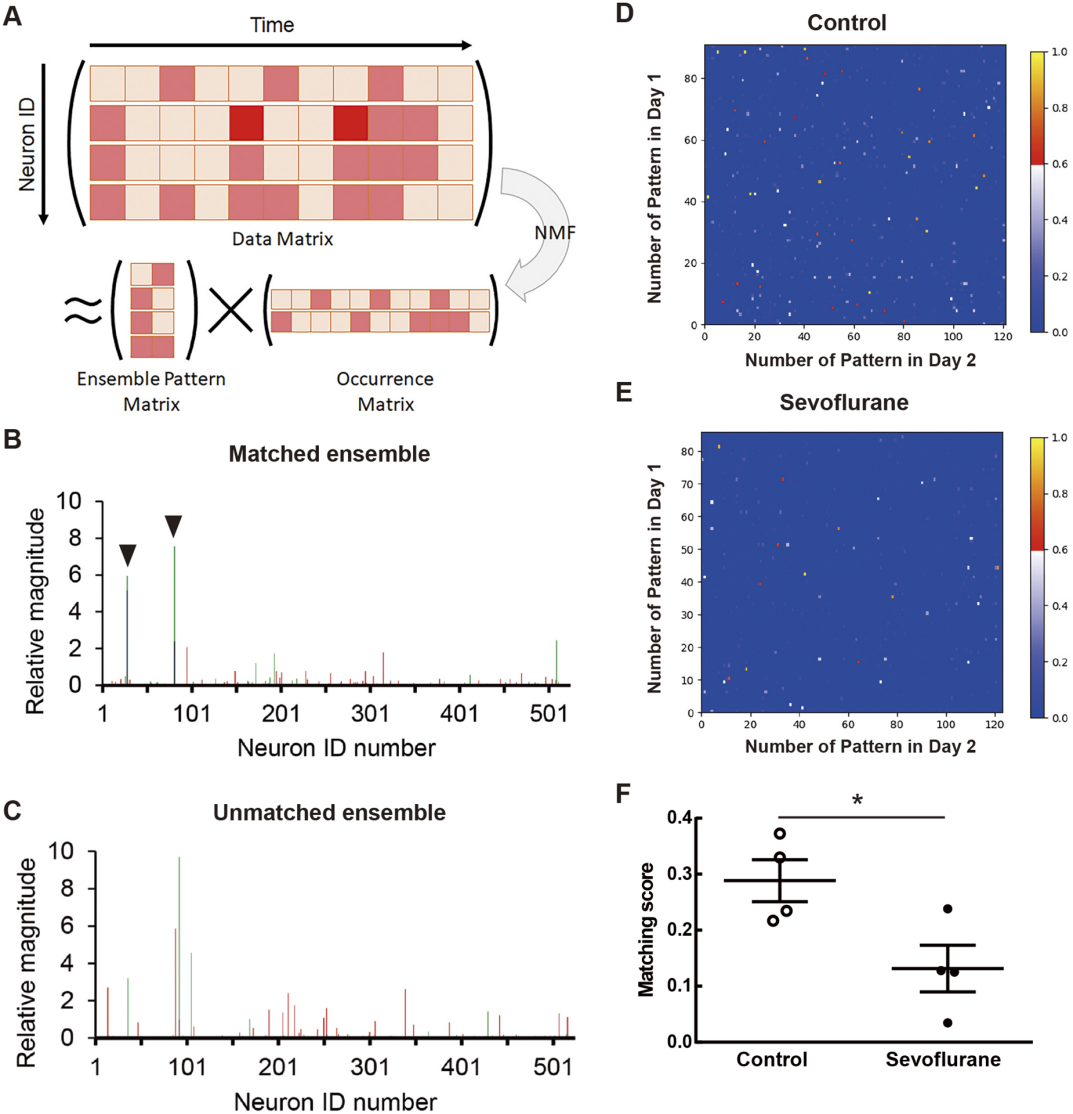
**Sevoflurane suppressed reactivation of neuronal ensembles detected in the Day 1 context session during Day 2 context session.** (A) Non-negative matrix factorization (NMF) analysis. (B,C) Representative neuronal ensemble patterns, including similar (B: dot product value = 0.704) and dissimilar (C: dot product value = 0.105) ensembles. Red and green bars mean an ensemble from Day 1 context and Day 2 context session. Arrow heads mean matched neurons between ensembles. (D,E) Representative images of cosine similarity of all ensemble pattern pairs between Day 1 context and Day 2 context sessions (C: non-anesthesia control, D: sevoflurane treatment). (F) Matching score of neuronal ensembles in Day 2 context session compared to Day 1 context session. (F) Non-anesthesia control mice (0.288 ± 0.037) versus sevoflurane treatment mice (0.131 ± 0.042), 95% CI = −0.294 to 0.020; *F*_(3,3)_ = 1.238, *P* = 0.8646; t_6_ = 2.803, *P* = 0.0310, unpaired *t*-test. *P < 0.05. Data are shown as means ± s.e.m.

For dissecting neuronal activity at ensemble level, we applied non-negative matrix factorization (NMF) analysis to whole neuronal activity data of each session to extract neuronal ensembles (Asai et al., 2020; Ghandour et al., 2019) as shown in Fig. 3A. Some ensembles detected in a session (e.g. Day 1 context) were observed in different session (e.g. Day 2 context) as matched ensembles that shared similar neuronal components (arrow heads, Fig. 3B) across sessions. These matched ensembles mean reactivation of the ensembles in different session. The other ensembles were observed in only the detected sessions (e.g. Day 1 context or Day 2 context) as unmatched ensembles which did not share similar neuronal components each other (Fig. 3C). These unmatched ensembles were not activated in the other sessions. To quantify the similarity between neuronal ensembles in different sessions, the cosine similarity (normalized dot product) between the ensemble pattern vectors was calculated. In this calculation, the value of similarity ranges from 0 (completely different) to 1 (completely the same). The vertical and horizontal axes of the figure showed the number of patterns in each session and the dot colors showed the value of similarity between patterns in each session (Fig. 3D,E). Compared to the mice of sevoflurane group, the control mice had many ensemble patterns with high values of similarity between Day 1 context and Day 2 context sessions (Fig. 3D,E). We calculated a matching score (MS) between the Day 1 and Day 2 context sessions from the dot product similarity (Fig. 3F). The MS indicates the proportion that neuronal ensembles detected in the Day 1 context session are reactivated in Day 2 context session (Ghandour et al., 2019). The control mice had higher MS than the sevoflurane mice [control group (0.288 ± 0.037) versus sevoflurane group (0.131 ± 0.042), 95% confidence interval (CI) = −0.294 to 0.020; *P* = 0.031, unpaired *t*-test]. This shows that sevoflurane treatment reduced the reactivation of ensembles during context exploration between just before and one day after sevoflurane inhalation. No significant difference was observed in the number of detected cells and neuronal ensembles between the control and sevoflurane groups (Fig. S1). Furthermore, when ensemble pattern similarity between Day 1 context, rest, and Day 2 context sessions in control mice were quantified as the same as Fig. 3, Day 1 context ensembles reactivated in rest session tend to be more activated in Day 2 context session than non-reactivated ensembles (Fig. S2). This support our hypothesis that reactivation just after context learning is important for memory consolidation. Taken together, these findings suggest that the suppression of ensemble reactivation by sevoflurane could be a part of the cause retrograde amnesia.

## DISCUSSION

This study showed that sevoflurane treatment just after context learning impaired CPFE memory performance and that general anesthesia by sevoflurane inhibited neuronal activity in hippocampal CA1. Furthermore, reactivation of neuronal ensembles in exploring context across days was reduced by sevoflurane treatment just after first context exploration compared to non-anesthesia control. These results suggest that sevoflurane induced retrograde amnesia in hippocampus-dependent memory task because the anesthetics disrupts the memory consolidation of the context information acquired just before sevoflurane inhalation.

Our behavioral results suggested that sevoflurane can induce retrograde amnesia. This result is consistent with previous reports showing that anesthesia leads to retrograde amnesia (Gerlai and McNamara, 2000; O'Gorman et al., 1998). Our Ca^2+^ imaging data showed that sevoflurane inhibited neuronal activity in hippocampal CA1 during sevoflurane administration but the inhibition of neuronal activity did not continue next day. These data support that memory impairment by sevoflurane was induced by retrogradely inhibiting context memory consolidation in Day 1 rather than by anterogradely disturbing association learning in Day 2.

Our findings of imaging experiments suggest that sevoflurane-induced amnesia could be caused by the suppression of neuronal activity in the hippocampus just after learning. Previous studies have shown that general anesthesia prevents neural activity (Perouansky et al., 2010; Yang et al., 2021; Yin et al., 2016). Artificial inhibition of neuronal activity in post-learning period disrupts memory consolidation (Boyce et al., 2016; Kumar et al., 2020; Miyamoto et al., 2016). Our data showed that sevoflurane-treated mice were inhibited neuronal activity in hippocampal CA1 (Fig. 2). The inhibition of neuronal activity by general anesthesia during post-learning period can induce amnesia. Previous study reports that memory trace ensembles are activated during learning, are reactivated during the early period after learning, and then are reactivated in subsequent test session. In contrast, non-reactivated ensembles during the early post-learning period are poorly reactivated in the test session (Ghandour et al., 2019). Our data also showed similar ensemble dynamics that neuronal ensembles reactivated during rest period were reactivated in Day 2 context (Fig. S2). Thus, anesthesia during post-learning periods inhibits reactivation of activated ensembles, resulting in memory impairment.

We observed the effect of sevoflurane on hippocampal-dependent memory and neuronal ensemble activity in hippocampus, but many open questions remain: how much anesthetic can induce amnesia, how long the effect of anesthetics can be retroactive, what kinds of memory are affected, how sevoflurane affected hippocampal activity, and which are key brain regions. Moreover, isoflurane does not induce retrograde amnesia (Dutton et al., 2002), which suggest that different kind of anesthesia works at different sites of cells and brain region. Further research to answer these questions will help to understand the mechanism of anesthesia and proper usage of anesthesia.

In conclusion, post-learning sevoflurane inhibits the reactivation of neuronal ensembles in hippocampal CA1, changes ensemble reactivation during context exploration, and induces amnesia. Our findings provide insight into the mechanisms of retrograde amnesia by anesthesia but also significance about information processing under idling state and subconscious conditions.

## MATERIALS AND METHODS

### Animals

All procedures involving animals were approved by the Animal Care and Use Committee of the University of Toyama. C57BL/6J mice and c-fos-tTA mice were purchased from Japan SLC and the Mutant Mouse Regional Resource Centre (stock number: 031756-MU), respectively. Thy1-G-CaMP7-T2A-DsRed2 (Thy1-G-CaMP7) mice have been described previously (Ghandour et al., 2019; Sato et al., 2018 preprint). Naive mice were wild type C57BL/6J without surgery. All surgery was performed on male Thy1-G-CaMP7 mice or Thy1-G-CaMP7; c-fos-tTA double transgenic mice with a C57BL/6J background. The mice were maintained on a 12 h light:dark cycle (light on 7:00 a.m.) at 24 ± 3°C and 55 ± 5% humidity. The animals were given food and water *ad libitum*, as described previously (Ghandour et al., 2019).

### Behavioral analysis

Male naive mice (10-12 weeks old) were housed individually for at least 7 days before the behavioral experiment. These mice were trained using the context pre-exposure facilitation effect (CPFE) paradigm (Fanselow, 1990; Ohkawa et al., 2015; Rudy and O'Reilly, 2001; Schiffino et al., 2011; Suzuki et al., 2022) to investigate the effect of sevoflurane on hippocampal-dependent memory function. This experiment consisted of pre-exposure learning; rest or sevoflurane treatment; conditioning; and a test session (Fig. 1A). All procedures were performed during the light cycle.

On Day 1, both sevoflurane-treated and control groups of mice were pre-exposed to a context for 6 min (pre-exposure learning). Immediately after the pre-exposure learning, sevoflurane-treated group was anesthetized and the control group was returned to their home cage to rest. Sevoflurane-treated group was anesthetized with 2.5% sevoflurane (Wako Junyaku Kougyou, Osaka, Japan), which is equals to 1 minimum alveolar concentrate sevoflurane in mice (Flecknell, 2015), and carrier oxygen (2 L/min continuously for 30 min) in an anesthesia box. Afterwards, the mice were returned to their home cage. Control mice were returned to their home cage until the next day. On Day 2, both groups of mice received a foot shock (0.8 mA for 3 s) immediately after entering in the same context as pre-exposure learning (conditioning) and were then immediately returned to their home cages. On Day 3, both groups were exposed to the same context for 3 min to measure freezing behavior, which was evaluated using a video tracking system (Muromachi Kikai, Tokyo, Japan) as described in previous studies (Abdou et al., 2018; Asai et al., 2020; Ghandour et al., 2019; Ohkawa et al., 2015; Oishi et al., 2019; Suzuki et al., 2022).

The behavioral equipment was described previously (Asai et al., 2020; Ghandour et al., 2019; Ohkawa et al., 2015; Oishi et al., 2019). The context was a square box with a Plexiglass front, gray sides, and a back wall (width: 175 mm × depth: 165 mm × height: 400 mm; Fig. 1B). The floor had 26 stainless-steel rods connected to a shock generator (Muromachi Kikai, Tokyo, Japan). The overhead room lights lit the context, and background noise was provided by a fan inside the room. The equipment was cleaned with 80% ethanol before each experiment. The anesthesia box (Fig. 1C) was a square transparent box (width: 160 mm × depth: 260 mm × height: 150 mm) with two ports for gas flow. Sevoflurane gas and the carrier oxygen gas were carried by an SN-487-1 anesthesia machine (Shinano Seisakusho, Tokyo, Japan). The animals contained in their home cages were transferred from the housing to the front room, adjacent to the housing and experimental rooms, at least 10 min before the pre-exposure learning, conditioning, and test sessions in the experimental room.

### Surgery

All surgery was performed on male Thy1-G-CaMP7 mice or Thy1-G-CaMP7; c-fos-tTA mice. The mice were anesthetized by intraperitoneal injection of pentobarbital (Somnopentyl; Kyoritsu Seiyaku, Tokyo, Japan) as dose of 64.8 mg/kg of body weight or combination anesthetic [medetomidine hydrochloride (Domitor; Nippon Zenyaku Kogyo, Koriyama, Japan), 0.75 mg/kg of body weight, midazolam (Sandoz K. K., Tokyo, Japan) 4 mg/kg of body weight, and butorphanol tartrate (Vetorphale; Meiji Seika Pharma, Tokyo, Japan) 5 mg/kg of body weight)] as described previously (Asai et al., 2020; Ghandour et al., 2019; Kawai et al., 2011; Ohkawa et al., 2015; Oishi et al., 2019). The mice were then placed in a stereotactic apparatus (Narishige, Tokyo, Japan). Implantation of a gradient refractive index (GRIN) relay lens was performed as described previously (Asai et al., 2020; Barretto et al., 2011; Ghandour et al., 2019; Ghosh et al., 2011; Kitamura et al., 2015; Ziv et al., 2013). A 2.0 mm diameter craniotomy was made for a cannula lens sleeve (1.8 mm OD and 3.6 mm in length; Inscopix, Palo Alto, CA, USA). A part of neocortex and corpus callosum above the alveus were aspirated cylindrically using a 27-gauge dull needle (handmade) under constant irrigation with saline (Otsuka Pharmaceutical, Tokyo, Japan). The cannula lens sleeve was softly placed on the surface of the alveus and fixed to the edge of the craniotomy part with melted bone wax (Tokyo M. I. company, Tokyo, Japan) by low-temperature cautery (Bovie Medical, Clearwater, FL, USA). The center of the cannula lens sleeve was positioned at the right hippocampus (2.0 mm posterior to bregma, 1.5 mm lateral to bregma). Four pairs of anchor screws (total 8 anchor screws, Eicom) were fixed to the front, right, left, and back of the skull. The anchor screws were covered with dental cement (Province, Shofu, Kyoto, Japan) to fix the cannula lens sleeve to the skull. The animals anesthetized with the combination anesthetic were given an intraperitoneal injection of atipamezole (Antisedan; Nippon Zenyaku Kogyo, Koriyama, Japan) at a dose of 0.75 mg/kg, which is an antagonist of medetomidine, to promote recovery from the anesthesia. After the surgery to implant the cannula lens sleeve, the mice were housed individually until Ca^2+^ imaging.

More than 2 weeks after surgery, the mice were anesthetized as described above and placed in the stereotactic apparatus. A GRIN lens (1.0 mm outer diameter and 4.0 mm length; Inscopix, Palo Alto, CA, USA) was inserted into the cannula lens sleeve and fixed with ultraviolet-curing adhesive NOA 81 (Norland, Cranbury, NJ, USA). A microscope baseplate (Inscopix, Palo Alto, CA, USA) that was attached to an integrated miniature microscope (nVista^™^ HD 3; Inscopix, Palo Alto, CA, USA) was placed above the GRIN lens to allow observation of G-CaMP7 fluorescence and blood vessels in hippocampal CA1. The microscope baseplate was fixed to the anchor screws of the skull using dental cement. After the miniature microscope was detached from the baseplate, the GRIN lens was covered by attaching the microscope baseplate cover (Inscopix, Palo Alto, CA, USA) until Ca^2+^ activity was recorded. Atipamezole was administered as described above.

### Recording Ca^2+^ activity in freely moving mice

The mice were attached to the integrated miniature microscope in their home cage for around 30 min for 3 days to habituate them to the miniature microscope attachment. On the next day, the mice were attached to the miniature microscope in their home cage for around 10 min before imaging. Then, the mice were introduced to a novel context (Day 1 context) for 6 min, during which time Ca^2+^ activity was recorded (Fig. 2A). The mice were transferred to the anesthesia box or home cage while leaving the miniature microscope attached immediately after the 6-min context exposure. The sevoflurane-treated group was anesthetized continuously with 2.5% sevoflurane and oxygen (2 L/min for 5 min) in the anesthesia box and then for 25 min by the anesthesia mask. After the 30-min sevoflurane treatment, the mice were returned to their home cages, and the miniature microscope was detached. The mice in the control group were returned to their home cages just after context exposure (Day 1 context), and their Ca^2+^ activity was recorded for 30 min. After this 30 min recording, the miniature microscope was detached. One day later, all mice were exposed to the same context for 3 min, and the Ca^2+^ activity was recorded (Day 2 context). Ca^2+^ imaging was performed during the light cycle. Imaging movies were acquired with nVista acquisition software (ver. 3.0, Inscopix, Palo Alto, CA, USA) at 20 frames/s with the complementary metal oxide semiconductor sensor at an exposure time of 50 ms, a gain of 5/7, and light-emitting diode power of 1.2 mW/mm^2^.

The context and anesthesia boxes were the same as those described in the behavioral experiment. The anesthesia box had a slit for the cable of the miniature microscope.

### Ca^2+^ imaging data processing

The Ca^2+^ transients that were captured at 20 frames/s with the nVista acquisition software were processed basically as previously described (Aly et al., 2022; Asai et al., 2020; Ghandour et al., 2019; Kitamura et al., 2015; Nomoto et al., 2022 preprint; Wally et al., 2021 preprint). The Ca^2+^ imaging movies of the context session on Days 1 and 2 and of the rest session or sevoflurane treatment session on Day 1 were first pre-processed by Inscopix Data Processing Software (IDPS; ver 1.3.1.2796, Inscopix, Palo Alto, CA, USA). These movies were spatially downsampled by a factor of 2, and then motion artifacts were roughly corrected using the algorithm included in the software. The resulting movies were then processed using Mosaic software (Inscopix, Palo Alto, CA, USA), as described previously (Asai et al., 2020; Ghandour et al., 2019; Kitamura et al., 2015; Nomoto et al., 2022 preprint; Wally et al., 2021 preprint). Motion correction was performed [correction type: Translation+Skewing, spatial mean (*r* = 20 pixels) subtracted and spatial mean applied (*r* = 5 pixels)] using blood vessels as a landmark to maintain the same field of view and to correct for motion artifacts. Next, the movies were processed using Fiji software (Schindelin et al., 2012) (a distribution of ImageJ; ImageJ ver.1.52i, Java 1.8.0_66; National Institutes of Health, Bethesda, MD, USA). Each session movie was low-pass filtered (*r* = 20 pixels) and cropped at the same coordinates in each mouse. The fluorescence intensity change (Δ*F*(*t*)/*F*0 = (*F*(*t*)−*F*0)/*F*0) was calculated using the Mosaic software, where *F*(*t*) is the fluorescence intensity from an individual time frame of the movie and *F*0 is the mean fluorescence for the entire movie for that session.

### Cell Identification and Mathematical Analysis

For the analysis of neural activity during rest or sevoflurane treatment (Fig. 2), the movies of the context session and rest or sevoflurane treatment session on Day 1 and the Day 2 context session were concatenated using the Mosaic software to create a single movie of all sessions. To identify neural signals, 20 cellular activities were manually detected at random using the Fiji software. For the quantification of neural activity, Ca^2+^ events were counted to satisfy the following conditions: an activity >0.01 arbitrary unit (a.u.) and >3 standard deviation (SD) for the entire session.

To analyze the similarity of neuronal ensembles between the context sessions, the Day 1 and Day 2 context session movies were concatenated using Mosaic software (Fig. 3). Neural signals were identified using constrained non-negative matrix factorization for microendoscope data (CNMF-E; https://github.com/zhoupc/CNMF_E) (Zhou et al., 2018) which was applied to the concatenated movie in MATLAB (The MathWorks, Natick, MA, USA), as previously described (Ghandour et al., 2019). The CNMF-E output Ca^2+^ data matrix which represents Ca^2+^ activity in each time frame for every cell. To remove the low frequency fluctuation and background noise, output Ca^2+^ data was subjected to a high-pass filtering with 0.01 Hz cutoff and z score calculation from mean of each session, and negative z score was replaced to zero.

To extract neuronal ensemble activity patterns from the whole Ca^2+^ data matrix, non-negative matrix factorization (NMF) was applied to the data matrix in each session as described previously (Asai et al., 2020; Lee and Seung, 2001; Ghandour et al., 2019). Briefly, the Ca^2+^ data matrix (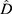; time × neuron) obtained by CNMF-E was binned every four frames (200 ms), and then NMF was applied. Consequently, 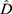 was optimally factorized into a basis matrix (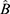; neuronal ensemble pattern matrix, ensemble × neuron) and the corresponding occurrence matrix (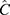; occurrence matrix, time × ensemble), 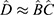. The Akaike information criterion with a second-order correction was used to determine the optimal number of ensembles. To find the optimal factorization, the ensemble (basis) matrix and intensity (occurrence) matrix that minimized the cost function defined as [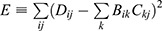] was chosen to be the optimal factorization when random initial entries from matrices 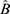 and 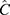 were used for 1000 attempts at minimization.

To quantify the similarity of ensembles across sessions, a matching score (MS) was calculated as described previously (Ghandour et al., 2019). The overall similarity between ensemble pattern vectors in two sessions X and Y was measured according to the normalized dot product, 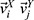, for all possible pattern pairs across the two sessions. Note that the dot product is equivalent to the cosine of the angle between the pattern vectors. Therefore, the MS between sessions X and Y is defined as MS (X, Y) 
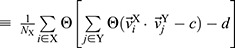
, where 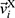 (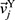) is the *i*th (*j*th) pattern vector in session X (Y), *N*_X_ is the number of pattern in session X, and Θ(·) is a step function. The constant *d* is an arbitrary positive number smaller than 1. This scoring function yields the portion of patterns in session X that have a normalized dot product larger than *c* with any of the patterns in session Y. A threshold of *c* = 0.6 was used throughout this study (Ghandour et al., 2019).

The reactivation of neuronal ensembles across the Day 1 context, Day 1 rest, and Day 2 context sessions were analyzed (Fig. S2). The movies of the Day 1 context session, first 1 min of rest, and Day 2 context session were concatenated. This concatenation was then followed by cellular identification using CNMF-E, extraction of neural ensembles using NMF, and quantification of ensemble similarity based on normalized dot products as described above. The percentages of reactivated patterns in test were calculated as follows: (the number of reactivated ensembles in Day 1 rest and Day 2 context sessions) / [the number of reactivated (or non-reactivated) ensembles in Day 1 rest session].

The source codes for NMF, cosine similarity, and MS are available on GitHub: https://github.com/IdlingBrainUT/NMF_Python.

### Statistical analysis

Statistical power calculation was not conducted before the study, because the sample sizes were determined based on previous experience with similar experimental protocols. The mice were randomly assigned to a sevoflurane treatment or non-anesthesia control group. Blinding methods were not used in the analysis of the behavioral and Ca^2+^ imaging experiments.

Statistical analyses were performed using GraphPad Prism^™^ 6 (GraphPad Software, San Diego, CA, USA) and MATLAB^®^ (The MathWorks, Natick, MA, USA). Data analyses were performed using an unpaired *t*-test, paired *t*-test, Welch's *t*-test, and a Bonferroni test for multiple-comparisons. A *P*-value of  < 0.05 was considered significant. Two-tailed comparisons were used in all comparison tests whenever the difference between the two groups was expected to be in either direction. Quantitative data are expressed as the mean ± standard error of mean (s.e.m.).

## Supplementary Material

10.1242/biolopen.059666_sup1 Supplementary information
