# Supplementary material for: Sevoflurane-induced amnesia is associated with inhibition of hippocampal cell ensemble activity after learning
Source: Biol Open. 2022 Dec 21;11(12):bio059666. doi: 10.1242/bio.059666 (PMC9793868; doi:10.1242/bio.059666)
Supplement: Supplementary information [file biolopen-11-059666-s1.pdf]

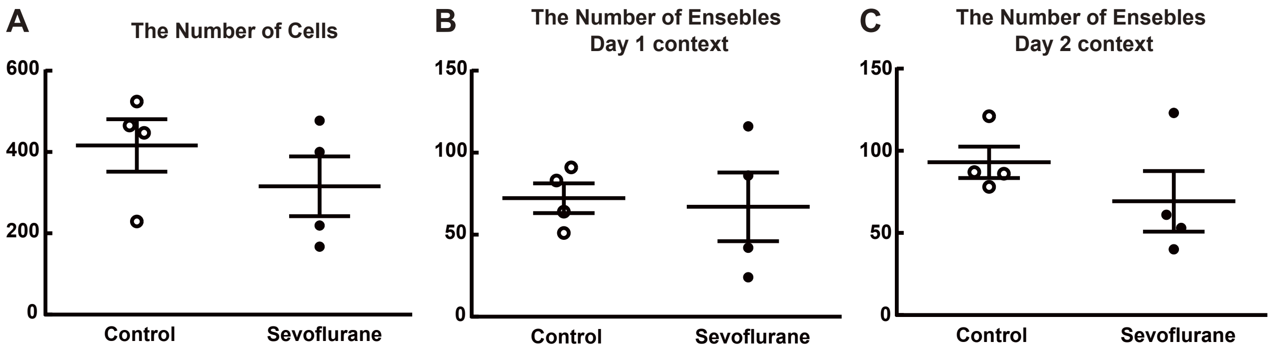

Fig. S1. No significant difference was observed in the number of detected neuronal cells and ensembles between the control and sevoflurane groups. (A) The number of cells detected during Day 1 and 2 context sessions. Non-anesthesia control mice [416.3 ± 64.55] vs. sevoflurane treatment mice [315.8 ± 73.36], 95% CI = -339.6 to 138.6; $F(3, 3) = 1.292$, $P = 0.8383$; $t6 = 1.028$, $P = 0.3434$, unpaired t-test. (B) The number of neuronal ensembles detected in Day 1 context session. Non-anesthesia control mice [72.25 ± 9.068] vs. sevoflurane treatment mice [67.00 ± 20.89], 95% CI = 60.97 to 50.47; $F(3, 3) = 5.306$, $P = 0.2039$; $t6 = 0.2305$, $P = 0.8253$, unpaired t-test. (C) The number of neuronal ensembles detected in Day 2 context session. Non-anesthesia control mice [93.00 ± 9.548] vs. sevoflurane treatment mice [69.25 ± 18.43], 95% CI = -74.54 to 27.04; $F(3, 3) = 3.726$, $P = 0.3086$; $t6 = 1.144$, $P = 0.2962$, unpaired t-test. Data are shown as means ± S.E.M.

Biology Open • Supplementary information

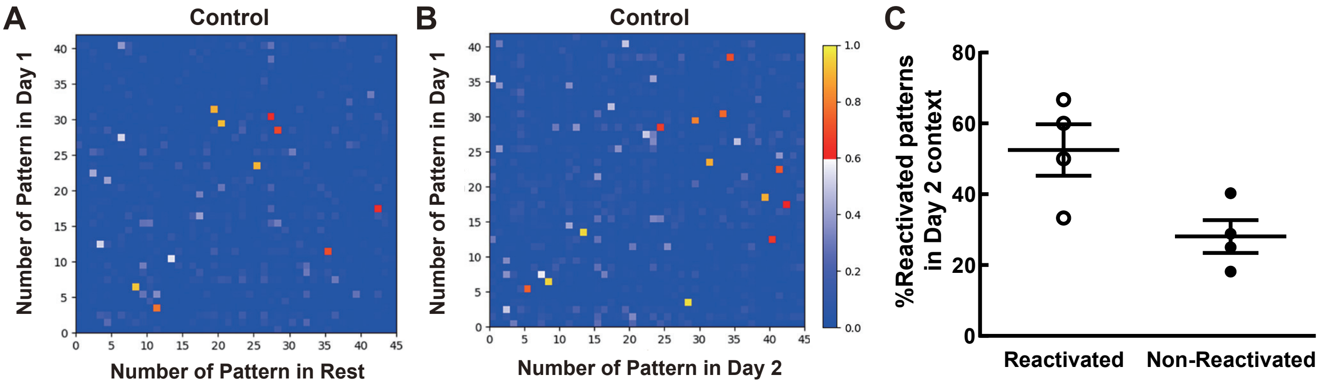

Fig. S2. Neuronal ensembles reactivated during the rest session tended to be more reactivated during test session in non-anesthesia mice. (A and B) Representative images of cosine similarity of all ensemble pattern pairs between Day 1 context, rest, Day 2 context sessions (A: Day 1 context and the rest session, B: the Day 1 context and Day 2 context session). (C) The proportion of neuronal ensembles reactivated in test session. Reactivated ensembles [52.50] vs. non-reactivated ensembles [28.09]; t3 = 2.549, P = 0.084, paired t-test. Data are shown as means ± S.E.M.

Table S1. Statistical data table for the behavioral experiment.

Session	Minute	Mean of control group	Mean of sevoflurane group	95% CI of difference	Adjusted *P*-value
Pre-exposure	1	0.000	0.000	-3.982, 3.982	> 0.9999
Fig. 1D	2	0.350	0.755	-4.386, 3.557	> 0.9999
	3	0.375	0.300	-3.907, 4.507	> 0.9999
	4	2.500	0.518	-2.000, 5.964	> 0.9999
	5	1.225	3.318	-6.075, 1.889	0.9617
	6	3.775	2.964	-3.171, 4.793	> 0.9999
Test	1	32.88	10.95	1.358, 42.50	**0.033***
Fig. 1E	2	30.41	19.61	-9.769, 31.38	0.5983
	3	25.15	19.97	-15.39, 25.75	> 0.9999
